# Adenovirus early region 3 RIDα protein limits NFκB signaling through stress-activated EGF receptors

**DOI:** 10.1371/journal.ppat.1008017

**Published:** 2019-08-19

**Authors:** Xuehuo Zeng, Cathleen R. Carlin

**Affiliations:** 1 Department of Molecular Biology and Microbiology, School of Medicine, Case Western Reserve University, Cleveland, United States of America; 2 Case Comprehensive Cancer Center, School of Medicine, Case Western Reserve University, Cleveland, United States of America; Stony Brook University, UNITED STATES

## Abstract

The host limits adenovirus infections by mobilizing immune systems directed against infected cells that also represent major barriers to clinical use of adenoviral vectors. Adenovirus early transcription units encode a number of products capable of thwarting antiviral immune responses by co-opting host cell pathways. Although the EGF receptor (EGFR) was a known target for the early region 3 (E3) RIDα protein encoded by nonpathogenic group C adenoviruses, the functional role of this host-pathogen interaction was unknown. Here we report that incoming viral particles triggered a robust, stress-induced pathway of EGFR trafficking and signaling prior to viral gene expression in epithelial target cells. EGFRs activated by stress of adenoviral infection regulated signaling by the NFκB family of transcription factors, which is known to have a critical role in the host innate immune response to infectious adenoviruses and adenovirus vectors. We found that the NFκB p65 subunit was phosphorylated at Thr254, shown previously by other investigators to be associated with enhanced nuclear stability and gene transcription, by a mechanism that was attributable to ligand-independent EGFR tyrosine kinase activity. Our results indicated that the adenoviral RIDα protein terminated this pathway by co-opting the host adaptor protein Alix required for sorting stress-exposed EGFRs in multivesicular endosomes, and promoting endosome-lysosome fusion independent of the small GTPase Rab7, in infected cells. Furthermore RIDα expression was sufficient to down-regulate the same EGFR/NFκB signaling axis in a previously characterized stress-activated EGFR trafficking pathway induced by treatment with the pro-inflammatory cytokine TNF-α. We also found that cell stress activated additional EGFR signaling cascades through the Gab1 adaptor protein that may have unappreciated roles in the adenoviral life cycle. Similar to other E3 proteins, RIDα is not conserved in adenovirus serotypes associated with potentially severe disease, suggesting stress-activated EGFR signaling may contribute to adenovirus virulence.

## Author summary

Although most adenovirus infections produce a mild and self-limiting disease, they can be life threatening for immunocompromised individuals. Some serotypes also cause epidemic outbreaks that pose a significant health risk in people with no known predisposing conditions. Although the early region 3 (E3) of the adenovirus genome is known to play a critical role in viral pathogenesis, experimental evidence regarding the molecular mechanisms effecting damage in the host is still limited. Here we provide the first studies showing that adenovirus infection induced stress-activated EGF receptor (EGFR) pro-inflammatory signaling prior to nuclear translocation and transcription of viral DNA in non-immune epithelial target cells. We have also identified host molecular mechanisms co-opted by the E3 RIDα protein that potentially limit immune-mediated tissue injury caused by stress-activated EGFR. There is increasing evidence that many viruses exploit EGFR function to facilitate their replication and antagonize host antiviral responses. Until now, it was generally assumed that viruses co-opted mechanisms induced by conventional ligand-regulated pathways. Recognition that stress-activated EGFR signaling may play a critical role in viral pathogenesis is significant because unique host proteins regulating this pathway represent novel drug targets for therapeutic development.

## Introduction

Human adenoviruses provide an excellent example of how viruses adapt host cell machinery to invade cells, gain access to the nucleus to replicate, assemble new viral particles, and spread in the host [1,2,3]. The host limits adenovirus infections by mobilizing innate immune systems that activate inflammatory or cytotoxic responses directed against infected cells [3,4,5,6]. These host defense mechanisms also represent a major barrier to the use of adenovirus vectors with many important clinical applications, ranging from cancer gene therapy to vaccine development [3,4,5,6]. In addition to specialized immune cells that secrete pro-inflammatory cytokines at sites of infection, immune and non-immune target cells both rely on cell autonomous innate immunity to defend against the immediate threat of infection [7]. Adenovirus circumvents various innate defense mechanisms by virtue of viral proteins encoded by early transcription units that strike a balance between the elimination of virus and immune-mediated tissue injury [8]. The study of cellular pathways used by viruses has led to many significant advances in eukaryotic cell and molecular biology [9]. Adenovirus early region 3 (E3) gene products in particular have been powerful tools for discovering new mechanisms in the fields of intracellular protein and lipid trafficking [10,11,12].

The adenovirus gene regulatory program involves two distinct phases during lytic infections. E3 transcripts are maximally expressed through transactivation by the early region 1A (E1A) gene product during the early phase, and subsequently repressed after the onset of DNA replication [13,14]. The E3 promoter also has NFκB binding sites that are highly sensitive to activation signals in human T cells in the absence of E1A, suggesting these proteins have key roles in persistently infected T lymphocytes [15]. The E3 region encodes several proteins that are specifically involved in immune evasion by modulating the function of cell surface receptors, intracellular signaling events, and secretion of pro-inflammatory molecules [11,16,17,18]. Many E3 proteins target host cell pathways with important roles in both immune and non-immune cells [11,16,17,18]. However, other host targets such as the EGF receptor (EGFR) are predominantly expressed in non-immune epithelial cells, suggesting E3 proteins also have cell-specific functions that have not been fully explored [19].

Although most adenovirus infections are self-resolving, some serotypes can lead to a serious and frequently fatal condition called acute respiratory distress syndrome [3,4,20]. Over the past few years adenoviruses have increasingly been recognized as significant pathogens associated with high morbidity and mortality in immune-compromised individuals, especially in the pediatric hematopoietic transplant population [21,22,23,24,25,26]. However, periodic adenovirus outbreaks can also pose significant health risks in people with no known predisposing conditions, such as US military personnel and civilians in closed community settings [27,28]. Understanding the cellular mechanisms targeted by adenoviruses is essential for developing new therapeutic approaches against viral diseases. In addition, common human diseases such as cancer and diabetes frequently manipulate the same cellular pathways as infectious agents [29,30]. In contrast to E1A and early region 1B (E1B) genes that are conserved among different adenovirus serotypes, E3 genes vary markedly and may even be absent, supporting their potential roles in adenovirus pathogenesis [26].

The inducible expression of pro-inflammatory cytokines under regulation of the nuclear factor kappa-light-chain-enhancer of activated B cells (NFκB) family of transcription factors is a key component of the host innate immune response to infectious adenoviruses and adenovirus vectors [3,4,31]. As with other viruses such as HSV-1, NFκB activation appears to occur in waves that presumably induce different patterns of gene expression over the course of an acute adenovirus infection [32]. The first wave is regulated by a rapid, transient mechanism involving PI3K (phosphoinositide 3-kinase)/Akt signaling, which is activated by the interaction between adenoviral capsids and host cell alpha (V) integrin receptors in pure cultures of alveolar epithelial cells [33,34,35,36,37]. A second wave has been linked to early gene expression. For instance, multiple lines of evidence indicate that E1A sensitizes infected respiratory epithelial cells to bacterially-derived LPS (lipopolysaccharide), which is present as a contaminant on airborne particles and activates NFκB following its binding to TLR4 (Toll-like receptor 4) [38]. A second example involves the E3-19K viral protein, which is known to trigger protein overload in the endoplasmic reticulum (ER) leading to calcium release and subsequent production of reactive oxygen intermediates mediating NFκB activation [39]. Mechanisms for switching between different modes of NFκB-dependent gene transcription are not currently known. It has also not yet been established how factors such as cellular stress shape the NFκB response to adenoviral infection [40,41].

Recent evidence indicates that a variety of cellular stresses or stress inducers stimulate a robust, non-canonical pathway of ligand-independent EGFR trafficking, frequently downstream of p38-MAPK activity [42,43]. Although these stress-induced EGFR responses are thought to provide cancer cells with a survival advantage and resistance to therapeutics, their potential roles during viral infections are largely unknown [42,43]. In contrast to ligand-stimulated counterparts that are targeted for degradation, stress-exposed EGFRs accumulate on both limiting membranes and intraluminal vesicles (ILVs) in a relatively stable population of multivesicular body (MVB) endosomes where they are subsequently activated [42,43]. ILVs appear to undergo dynamic cycles of fission and fusion at the limiting membrane, enabling resumption of EGFR stress signaling from MVB limiting membranes [44]. It is also thought that ILV back-fusion facilitates EGFR recycling back to plasma membrane when p38-MAPK activity declines [45]. A number of EGFR signaling pathways have been linked to NFκB activation downstream of ligand stimulation or constitutive EGFR activation in cancer cells, primarily by promoting degradation of inhibitor of kappa B (IkB) proteins that sequester inactive NFκB proteins in the cytoplasm [40,46]. However, there is little information regarding potential links between stress-induced EGFR signaling and NFκB.

We have reported previously that cell surface levels of EGFR and related tyrosine kinase family members were significantly reduced following acute infection with group C adenoviruses [19,47,48]. Using an extensive series of adenovirus deletion mutants, we mapped the viral gene responsible for this effect to an E3 transcript encoding a small membrane protein called RIDα-C2 [48,49]. We have also shown that RIDα-C2 was a resident membrane protein in a novel population of endosomes, where it transiently interacted with EGFRs and re-routed them for degradation [19,50,51,52]. In contrast to the ligand-induced pathway, however, adenovirus-induced EGFR trafficking did not require intrinsic tyrosine kinase activity or receptor ubiquitination [19,53]. However, the role of EGFR in adenovirus pathogenesis has remained elusive, due in part to an incomplete understanding of the underlying mechanism of RIDα-induced EGFR trafficking. Here we report two novel findings. First, adenovirus infection induced an EGFR stress response that generated a phosphorylated NFκB binding site for the peptidyl-prolyl isomerase Pin1, which is known to enhance NFκB activity by countering negative feedback control through ubiquitin (Ub)-mediated NFκB degradation [54]. Second, the adenoviral RIDα protein terminated this pathway by co-opting host machinery regulating sorting of stress-exposed EGFRs in MVB endosomes and promoting lysosome fusion. These studies have yielded new insights to the molecular basis of stress-induced EGFR trafficking and signaling, as well as unappreciated EGFR functions that may be exploited by other viruses.

## Results

### EGFR was down-regulated in acutely infected A549 cells with physiological levels of receptor and trafficking proteins

**Fig 1:**
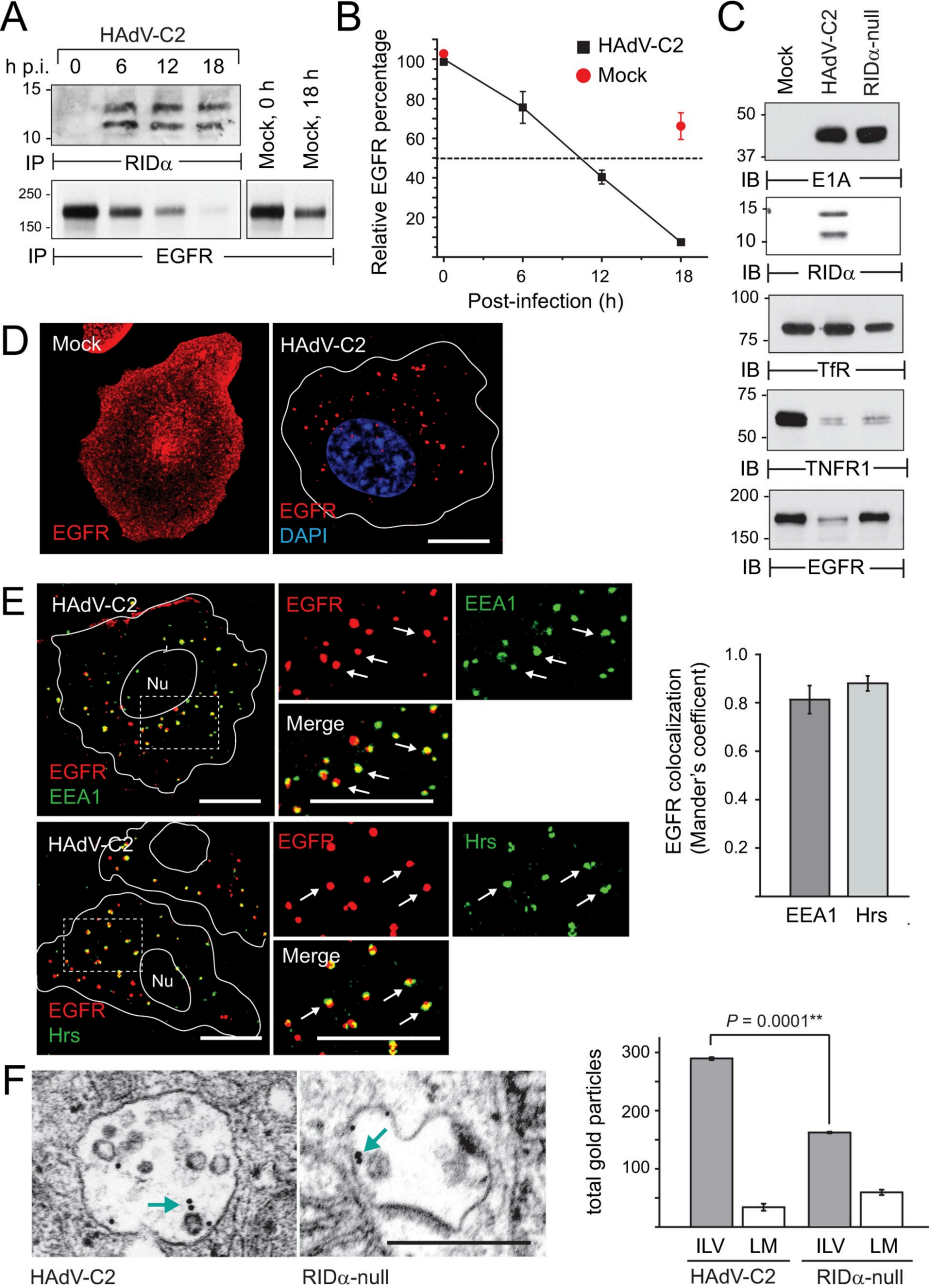
EGFR was associated with endosomal compartments in adenovirus-infected cells. (A) Mock-treated A549 cells and cells infected with HAdV-C2 (MOI = 100) were metabolically labeled from 1 to 2 h p.i., and RIDα (top panel) and EGFR (bottom panel) immune complexes were detected by fluorography every 6 h post-infection (p.i.). RIDα-C2 migrated as two molecular weight species because it has a partially cleaved amino-terminal ER signal sequence [49]. (B) EGFR metabolic half-life (dashed line) was determined by quantifying EGFR bands in (A), and representing data as the percentage of radioactive EGFR at time 0 in mock-treated and infected cells (red and black squares respectively) (mean ± s.e.m., n = 2). (C) A549 cells were mock-treated, or infected with HAdV-C2 or a RIDα-null HAdV-C2 mutant that deletes 107 base pairs in the amino terminal region of the viral protein [65] (MOI = 100). Cells were harvested 12 h later for immunoblot analysis with antibodies to virally-encoded proteins (E1A and RIDα) and host cell receptors (TfR, TNFR1, and EGFR). (D) Representative confocal images of mock-treated A549 cells or cells infected with HAdV-C2 (MOI = 100) for approximately 6 h, that were stained with EGFR antibody (red). (E) Representative confocal images of A549 cells infected with HAdV-C2 (MOI = 100) for 8 to 10 h stained with antibodies to EGFR (red) and EEA1 or Hrs (green). Individual and merged fluorescent signals are shown in high magnification images (2×) of boxed areas. Quantification of co-localization between EGFR and EE1A or Hrs is shown to the right of images. (D and E) All size bars, 5 μm. (F) Representative images showing MVBs from cells infected with HAdV-C2 or the RIDα-null mutant virus (MOI = 100) labeled with colloidal gold-conjugated EGFR antibody, for detection by transmission electron microscopy. Blue arrows highlight gold particles associated with MVBs; size bar, 5 μm. Quantification of the total gold particles per 50 cells localized on MVB ILVs or limiting membranes (LM) is shown to the right of the images (mean ± s.e.m., n = 2).

A majority of our previous studies analyzing the effect of adenovirus infection on EGFR trafficking were carried out in cancer cell lines and heterologous cell models with pathological levels of EGFR (> 10^6^ receptors/cell) [19,51,53,55]. In order to establish the role of host proteins in adenovirus-regulated EGFR trafficking, it was important to utilize cells with receptor expression closer to physiological levels to avoid saturation of the trafficking machinery [56,57,58]. We tested whether A549 lung epithelial cells were an appropriate tissue culture model for two main reasons. First, although this is a cancer cell line, A549 cells express wild-type (WT) EGFRs within the range of physiological receptor expression (~10^5^ receptors/cell) [55,59]. Second, they are a well-established airway epithelial tissue culture model for analyzing host responses to acute adenovirus infections and adenoviral-based therapeutics [35,60,61,62]. To determine their suitability for these studies, A549 cells were pulse-labeled with radioactive amino acids shortly after infection with HAdV-C2, and RIDα and EGFR proteins were recovered by immunoprecipitation for analysis by SDS-PAGE. We found that the viral protein reached steady-state expression by 6 h post-infection (p.i.) (Fig 1A). HAdV-C2 infection was also associated with a significant reduction in EGFR metabolic half-life, which typically ranges from 18 to 24 h under basal conditions [63,64] (Fig 1A and 1B). Total EGFR protein levels were compared by immunoblotting whole cell lysates from cells infected with HAdV-C2, versus a HAdV-C2 mutant with an amino-terminal 107 base pair deletion in the RIDα gene (labeled “RIDα-null”) [49,65]. Cells infected with either virus expressed equivalent levels of E1A, the first viral gene product expressed post-infection (Fig 1C). However, only cells infected with HAdV-C2 produced E3-encoded RIDα proteins (Fig 1C). Consistent with reports in the literature, HAdV-C2 infection was associated with a significant reduction in total tumor necrosis factor receptor (TNFR1) protein, but did not significantly alter total protein levels of the transferrin receptor (TfR) (Fig 1C). In contrast to TNFR1, infection with the RIDα-null virus was sufficient to block EGFR down-regulation independently of other virally-encoded proteins (Fig 1C). Confocal imaging revealed that EGFR accumulated in intracellular vesicles following infection with HAdV-C2, in contrast to mock-treated cells where EGFRs were predominantly associated with plasma membrane (Fig 1D). Internalized EGFRs were extensively co-localized with markers for early (EEA1 and Hrs) endosomes at 8 to 10 h post-infection, suggesting EGFRs trafficked to these compartments prior to degradation (Fig 1E). EGFR distribution on MVB limiting membranes and ILVs was examined by transmission electron microscopy in cells infected with HAdV-C2 or the RIDα-null mutant virus, that were stained with a colloidal gold-conjugated EGFR antibody (Fig 1F) [66]. Gold particles associated with MVB compartments were quantified in a total of 50 cells in two independent experiments. We found that MVB limiting membranes had similar EGFR content in cells infected with either virus, but that there was a statistically significant increase in the number of gold particles localized on MVB ILVs in cells infected with HAdV-C2 compared to the RIDα-null mutant virus (Fig 1F). Collectively these data established that RIDα promoted endolysosomal EGFR sorting at physiological levels of receptor expression in HAdV-C2 infected epithelial cells.

### EGFR trafficking was regulated by a subset of ESCRT machinery in HAdV-C2 infected cells

**Fig 2:**
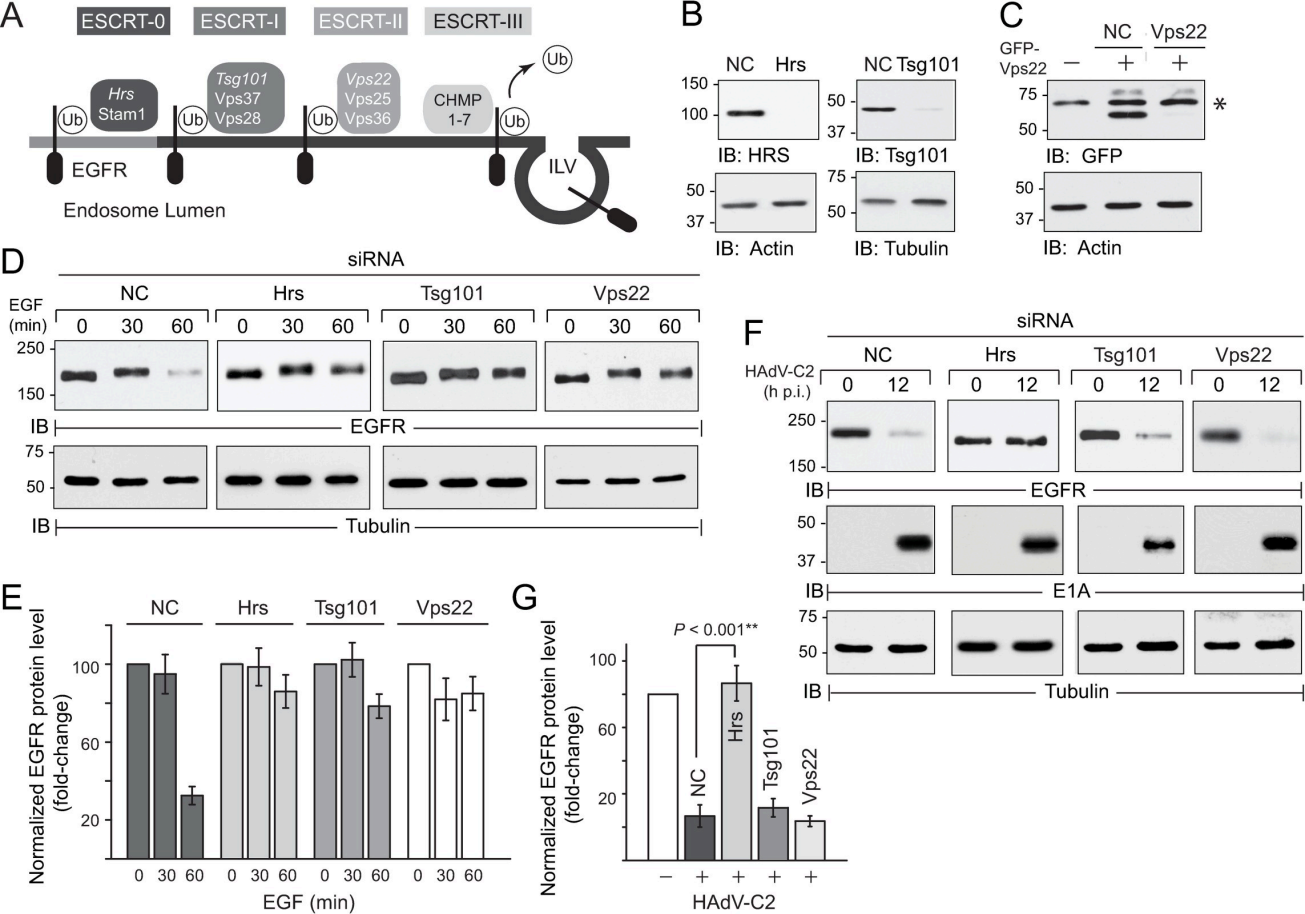
EGFR trafficking was regulated by a subset of ESCRT machinery in HAdV-C2 infected cells. (A) Simplified schematic of canonical ESCRT machinery that mediates MVB sorting of ubiquitinated (Ub) cargo including ligand-activated EGFR [128]. Key ESCRT subunit-specific siRNA targets are highlighted in *italics*. (B and C) ESCRT subunit knockdown was monitored by immunoblotting following treatment with non-coding (NC) or ESCRT-specific siRNAs for 72 h. Hrs and Tsg101 knockdown were assessed with antibodies to the native proteins (B), and Vps22 knockdown in cells transfected with a siRNA-sensitive GFP-Vps22 plasmid (C). The GFP antibody detected a non-specific protein (asterisk) in addition to GFP-Vps22 (C). (D and E) Equal protein aliquots of whole cell lysates (WCL) from siRNA-treated cells stimulated with EGF (50 ng/ml) up to 60 min were analyzed by immunoblotting for total EGFR protein (D). EGFR protein bands in (D) were normalized to the loading control for each sample, and data are presented as fold-change relative to the band at time 0 for each siRNA treatment (mean ± s.e.m., n = 3) (E). (F and G). Equal protein aliquots of WCL from siRNA-treated cells infected with HAdV-C2 (MOI = 100) for 12 h were analyzed for total EGFR protein or E1A by immunoblotting (F). Quantification of normalized EGFR protein bands in (F) are presented as fold-change relative to the band in uninfected cells for each siRNA treatment (mean ± s.e.m., n = 3) (G). (B–D, F). Filters were re-probed with antibodies to actin or tubulin to control for protein loading.

For the majority of membrane proteins that have been studied in detail including ligand-activated EGFRs, ILV sorting is regulated by the canonical ESCRT machinery targeting Ub-cargo (Fig 2A) [67]. Briefly, ESCRT-0 comprised of Hrs and Stam1 subunits sequesters ligand-stimulated receptors in peripheral early endosomes, and ESCRT-I and ESCRT-II act sequentially to sort Ub-EGFRs into inward invaginations of limiting membranes [68]. ESCRT-III then facilitates cargo deubiquitination and membrane scission finalizing ILV formation [69]. The molecular basis of the adenovirus-induced sorting pathway was determined by systematic depletion of key subunits *italicized* in Fig 2A, which are known to regulate the functional integrity of various ESCRT complexes, using targeted small interfering RNAs (siRNAs) (Fig 2B and 2C) [70]. Depletion of individual ESCRT subunits led to a significant reduction in ligand-induced EGFR down-regulation (Fig 2D and 2E), consistent with reports in the literature [70]. In contrast, the adenovirus-induced pathway was inhibited by reduced ESCRT-0 function, but ESCRT-I and ESCRT-II were dispensable (Fig 2F and 2G). The lack of phenotype was not due to differences in infectivity, since E1A proteins were similarly expressed in cells with reduced expression of individual ESCRT subunits (Fig 2F). These findings suggested that adenovirus-regulated EGFR trafficking had overlapping but distinct requirements for ESCRT machinery compared to the ligand-stimulated pathway. They are also consistent with previous results that receptor ubiquitination was not required for adenovirus-mediated EGFR trafficking [53].

### The adenovirus-induced EGFR trafficking pathway was regulated by Alix

**Fig 3:**
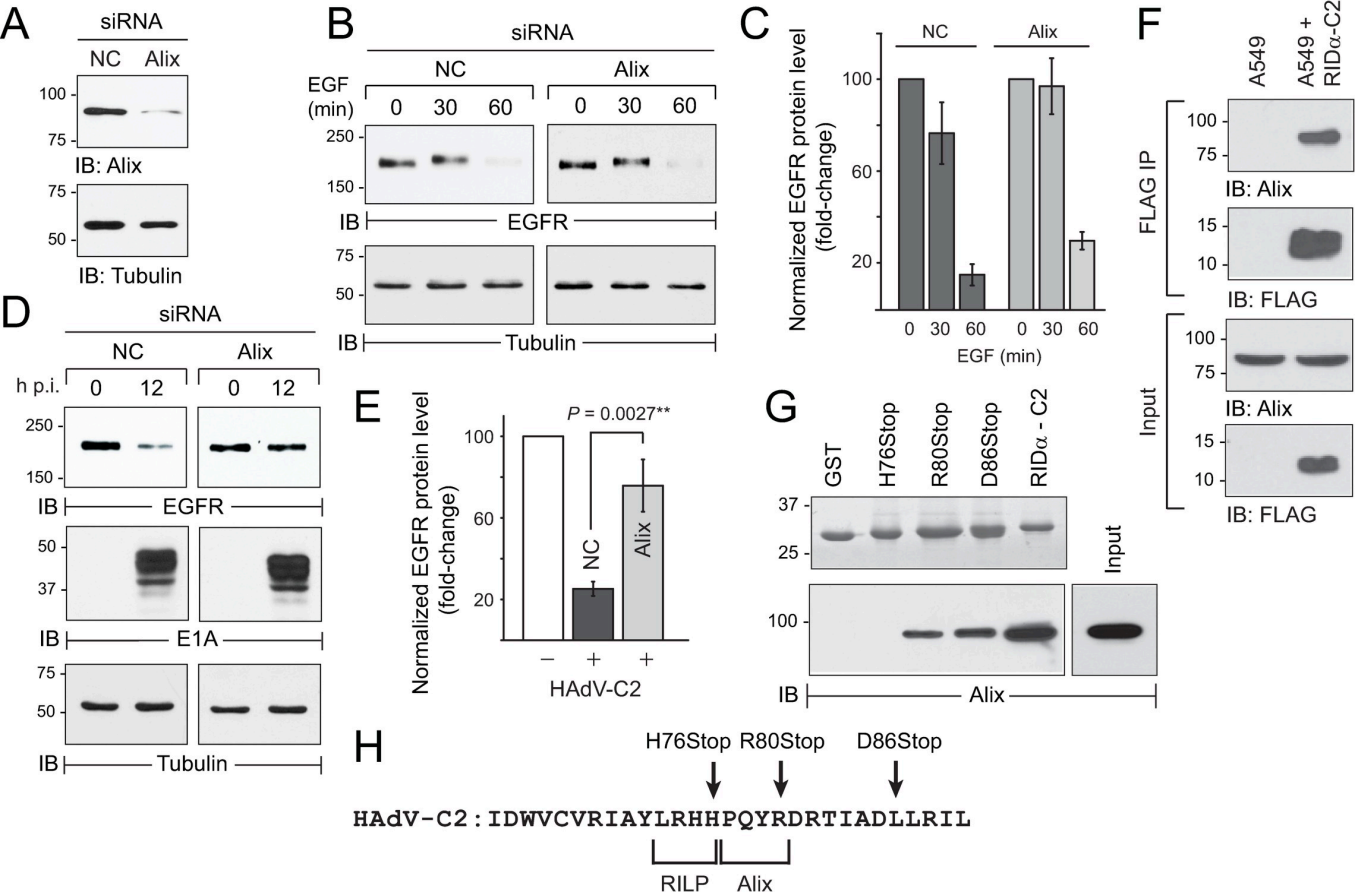
RIDα promoted EGFR down-regulation through an interaction with Alix. (A) Alix siRNA knockdown efficiency and tubulin protein loading controls were monitored by immunoblotting at 72 h post-transfection. (B and C) siRNA-treated cells were stimulated with EGF (50 ng/ml) to analyze total EGFR protein by immunoblotting (B). Quantification of normalized EGFR protein bands in (B) are presented as fold-change relative to the band at time 0 for each siRNA treatment (mean ± s.e.m., n = 3) (C). (D and E) siRNA-treated cells were infected with HAdV-C2 (MOI = 100) to analyze total EGFR and E1A protein levels by immunoblotting (D). Quantification of normalized EGFR protein bands in (D) presented as fold-change relative to the band at in uninfected cells for each siRNA treatment (mean ± s.e.m., n = 3) (E). (B and D) Filters were re-probed with a tubulin antibody to control for protein loading. (F) FLAG immune complexes from parent A549 cells and cells with stable expression of a FLAG-tagged HAdV-C2 RIDα protein (RIDα-C2) (top panels labeled “FLAG IP”), and equal aliquots of WCL (bottom panels labeled “Input”), were analyzed by immunoblotting with Alix and FLAG antibodies. (G) Top: GST fusion proteins expressing the intact 30-amino acid C-terminus of HAdV-C2 RIDα or truncated peptides with premature stop codons were purified from *E*. *coli*, and resolved by SDS-PAGE for detection by Coomassie staining. Bottom: RIDα GST fusion proteins attached to glutathione beads were incubated with CHO WCL with physiological levels of endogenous Alix (see “Input” lane), and bound proteins were eluted for immunoblot analysis with an Alix antibody. (H) HAdV-C2 RIDα C-terminal sequences highlighting stop codons used to generate GST fusion proteins for pull-down assays (arrows), and amino acids required for the interaction with Alix, which is located adjacent to a previously identified binding site for RILP [77].

The multi-adaptor protein Alix has recently emerged as a key component in alternative ILV sorting pathways because of its ability to simultaneously bind non-Ub cargo, and mediate ILV sorting via a direct protein-protein interaction with the ESCRT-III subunit CHMP4B [71]. The role of Alix in HAdV-C2 infected cells was evaluated by siRNA gene silencing, which reduced Alix expression by ~ 80% relative to a non-coding siRNA (Fig 3A). As reported by several other groups, Alix depletion did not have a significant impact on EGFR down-regulation following ligand stimulation (Fig 3B and 3C) [72,73,74,75,76]. In sharp contrast, Alix gene silencing effectively blocked EGFR degradation in the HAdV-C2 induced pathway, without impacting viral infectivity assessed by E1A expression (Fig 3D and 3E). We also showed that Alix was detected in RIDα immune complexes isolated from cells with physiological levels of endogenous Alix and stable expression of a FLAG-tagged RIDα protein encoded by HAdV-C2 (RIDα-C2), suggesting these two proteins interacted under physiological conditions in vitro (Fig 3F). This interaction was also evaluated by incubating whole cell lysates with GST fusion proteins containing the 30-amino acid cytosolic tail of RIDα-C2 and truncated RIDα-C2 peptides with premature stop codons (Fig 3G and 3H) [52,77]. Immunoblot analysis revealed that RIDα-C2 and Alix formed a molecular complex involving RIDα-C2 residues 77-PQYR-80, which were located adjacent to a previously described binding site for the Rab7 effector RILP (Rab7 interacting lysosomal protein) (Fig 3G and 3H) [77]. Collectively our data supported a working model that HAdV-C2 diverted EGFRs to a non-canonical degradative pathway, via a direct interaction between a proline-containing motif in the RIDα cytosolic tail and Alix. It was also notable that HAdV-C2 co-opted the ESCRT-0 subunit Hrs (Fig 2F and 2G) and Alix (Fig 3), since both of these ESCRT components are required for stress-induced EGFR trafficking [44].

### RIDα promoted lysosomal degradation of stress-activated EGFRs in the absence of other viral proteins

**Fig 4:**
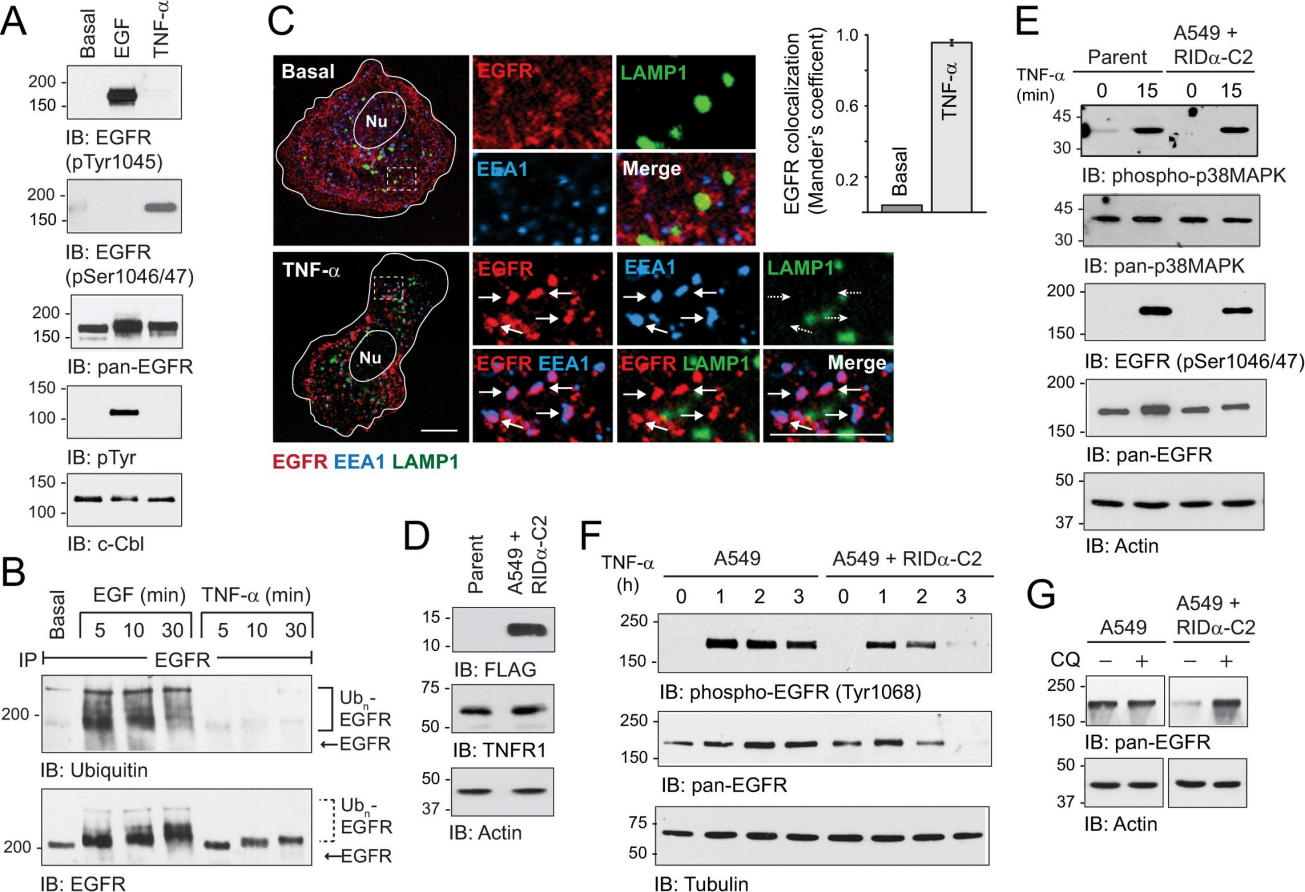
Stress-induced EGFR responses in A549 cell models. (A) A549 cells were harvested under basal conditions, or following 15-min stimulation with EGF or TNF-α (50 ng/ml each). Equal protein aliquots of WCL were immunoblotted with phospho-specific and pan EGFR antibodies (top 3 panels), and c-Cbl immune complexes were immunoblotted for phosphotyrosine and total c-Cbl protein (bottom 2 panels). (B) EGFR immune complexes from A549 cells were resolved on 5% SDS-PAGE gels to detect high molecular weight ubiquitinated EGFRs (Ub_n_-EGFR) following EGF or TNF-α stimulation (50 ng/ml each). Blots were probed with Ub-specific antibody and then stripped and re-probed for total EGFR protein. (C) Representative confocal images from A549 cells that were triple-stained with antibodies to EGFR (red), EEA1 (blue), and LAMP1 (green), under basal conditions or following 60-min stimulation with TNF-α (50 ng/ml). Individual and merged fluorescent signals are shown in high magnification images (4×) of boxed areas; size bars, 5 μm. Quantification of EGFR co-localization with EE1A under basal or TNF-α-stimulated conditions is shown to the right of the images. (D) Equal protein aliquots of WCL from parent A549 cells and A549 cells with stable HAdV-C2 RIDα expression (A549 + RIDα-C2) were immunoblotted with antibodies to FLAG-RIDα, TNFR1, and actin. (E) A549 cell models were harvested under basal conditions or following 15-min stimulation with TNF-α (50 ng/ml), for immunoblot analysis with phospho-specific and pan antibodies to p38-MAPK and EGFR, and actin to control for protein loading. (F) A549 cell models were analyzed under basal conditions, or following treatment with TNF-α (50 ng/ml). Equal aliquots of WCL were immunoblotted with phospho-specific and pan antibodies to EGFR and a tubulin protein loading control. (G). A549 cell models were treated with TNF-α (50 ng/ml) for 3 h under basal conditions, or following a 2-h pretreatment with chloroquine (CQ; 25 μM), for examination of total EGFR and actin protein levels by immunoblotting.

The hypothesis that RIDα modulated the stress-induced EGFR trafficking pathway was tested in cells treated with the pro-inflammatory cytokine TNF-α. The initial set of experiments confirmed that TNF-α elicited a typical EGFR stress response in A549 cells. We first showed that TNF-α induced EGFR phosphorylation at Ser1046/1047, which is known to mediate stress-induced EGFR internalization, in A549 cells (Fig 4A) [42,78]. In contrast, EGF stimulated EGFR phosphorylation at Tyr1045 that is the docking site for the E3 Ub ligase c-Cbl, which was also tyrosine phosphorylated in EGF-stimulated cells (Fig 4A). In addition, EGFR ubiquitination required in the canonical ESCRT pathway was induced by EGF but not TNF-α (Fig 4B). Finally, we demonstrated that internalized EGFRs accumulated in EEA1-positive early endosomes following 60-min stimulation with TNF-α (Fig 4C). EGFR stress responses were then compared in parent A549 cells versus cells with stable expression of FLAG-tagged RIDα-C2. We first showed that the viral protein did not have a significant impact on steady-state protein expression of the TNF-α receptor TNFR1 (Fig 4D). TNF-α also induced rapid p38-MAPK activation and EGFR phosphorylation at Ser1046/47 in parent A549 cells and cells with stable RIDα expression (Fig 4E). In addition, TNF-α stimulated EGFR activation in both cell models, as determined by immunoblotting with a phospho-specific EGFR antibody to the Tyr1068 autophosphorylation site (Fig 4F). However, autophosphorylated and total EGFR protein levels were markedly reduced within 2 to 3 h of TNF-α addition to RIDα-C2 expressing cells, compared to parent cells (Fig 4F). Furthermore, reduced EGFR expression observed in A549 + RIDα-C2 cells was blocked when cells were pretreated with the lysosomotropic agent chloroquine to inhibit protein degradation in lysosomes (Fig 4G) [79]. Altogether these results indicated that RIDα expression was sufficient to divert stress-internalized EGFRs to lysosomes.

### Stress-activated EGFR regulated NFκB-p65 through Thr254 phosphorylation

**Fig 5:**
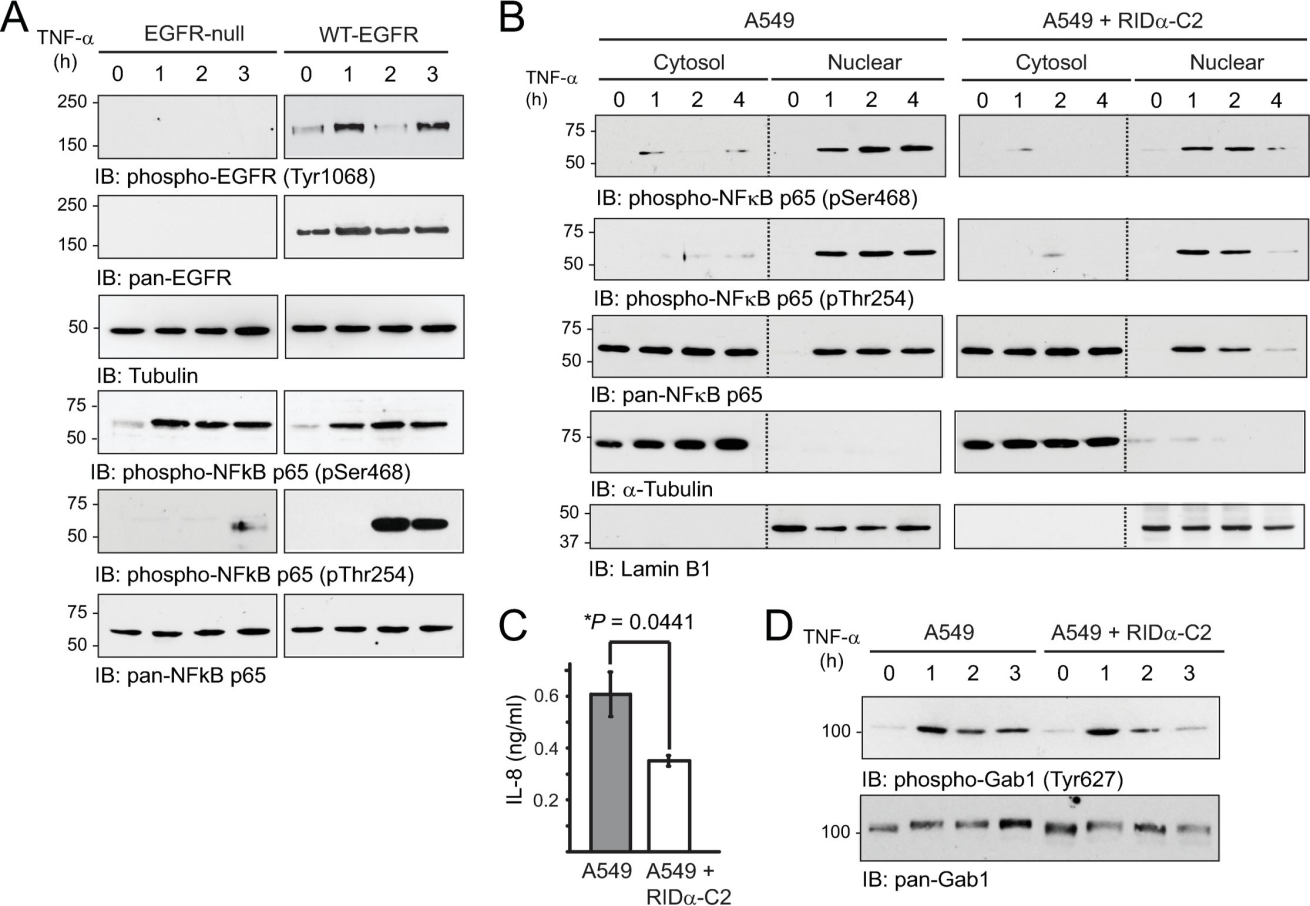
RID-α promoted EGFR degradation downstream of TNF-α. (A) EGFR-null mouse fibroblasts and EGFR-null cells reconstituted with WT-EGFR were analyzed under basal or TNF-α (50 ng/ml) treated conditions. Equal aliquots of WCL were immunoblotted with phospho-specific and pan antibodies to EGFR and NFκB-p65. (B) Equal aliquots of cytosolic and nuclear extracts prepared from A549 cell models under basal and TNF-α-stimulated conditions (50 ng/ml), were analyzed by immunoblotting with antibodies to NFκB (phospho-specific and pan), and cytosol (α-tubulin) and nuclear (lamin B) fractionation markers. (C) Tissue culture supernatants were analyzed for IL-8 protein by ELISA 24 hr after TNF-α stimulation (50 ng/ml) (mean ± s.e.m., n = 3). (D) Equal WCL aliquots from unstimulated and TNF-α-treated (50 ng/ml) A549 cell models were immunoblotted with phospho-specific and pan antibodies to Gab1. All experiments were repeated at least three times and representative results are shown.

Site-specific phosphorylation events play a critical role in the regulation of NFκB-p65 activity downstream of multiple stimuli, including TNF-α [80]. While many of the TNF-α-induced upstream signals have been identified, the pathway responsible for phosphorylation at the Thr254-Pro motif, which is a known substrate for the peptidyl-prolyl isomerase Pin1, was unknown [54]. We therefore used a genetic approach to investigate whether stress-activated EGFR contributed to phosphorylation at Thr254-Pro, by comparing responses in EGFR-null mouse fibroblasts versus cells that were reconstituted with elevated levels of wild-type (WT) human EGFR. We first showed that EGFR was autophosphorylated at Tyr1068 in response to TNF-α stimulation in the reconstituted cells (Fig 5A). The EGFR activation profile appeared to be biphasic, which would be consistent with re-activation of EGFRs that have recycled from ILVs back to MVB limiting membranes [42,43]. TNF-α stimulated phosphorylation at Ser468 located in the NFκB-p65 transactivation domain with similar kinetics and duration in both cell lines (Fig 5A). However, robust Thr254 phosphorylation was only observed in the EGFR-reconstituted cells (Fig 5A). It has already been established that Pin1-mediated prolyl isomerization leads to NFκB-p65 nuclear accumulation, increased NFκB-p65 protein stability, and enhanced transcription [54,81]. We therefore tested the hypothesis that stable RIDα expression would have a negative impact on TNF-α-induced NFκB-p65 signaling. In cell fractionation studies, we showed that Ser468-phosphorylated and Thr254-phosphorylated NFκB-p65 species were mainly localized in the nucleus, consistent with nuclear translocation, in both cell models (Fig 5B). However, nuclear levels of total and phosphorylated NFkB-p65 proteins were markedly reduced in cells expressing the RIDα-C2 protein (Fig 5B). We also examined production of the NFκB target interleukin-8 (IL-8) by ELISA of tissue culture supernatants (Fig 5C). The induction of IL-8 expression 24 h post-stimulation (~ 0.6 ng/ml) was on par with other reports for TNF-α treatment of A549 cells (e.g. [82]) (Fig 5C). Constitutive RIDα-C2 expression led to a significant reduction in secreted IL-8 protein (Fig 5C), consistent with attenuated NFκB-p65 gene transcription. Finally, we showed that stress-activated EGFR signaling through the adaptor protein Gab1 was also attenuated in the RIDα-C2 expressing cells compared to parent A549 cells (Fig 5D). These results suggested that the EGFR/NFκB pathway may be part of a broader stress-induced signaling network.

### Virus cell entry activated the EGFR/NFκB pathway induced by cellular stress

**Fig 6:**
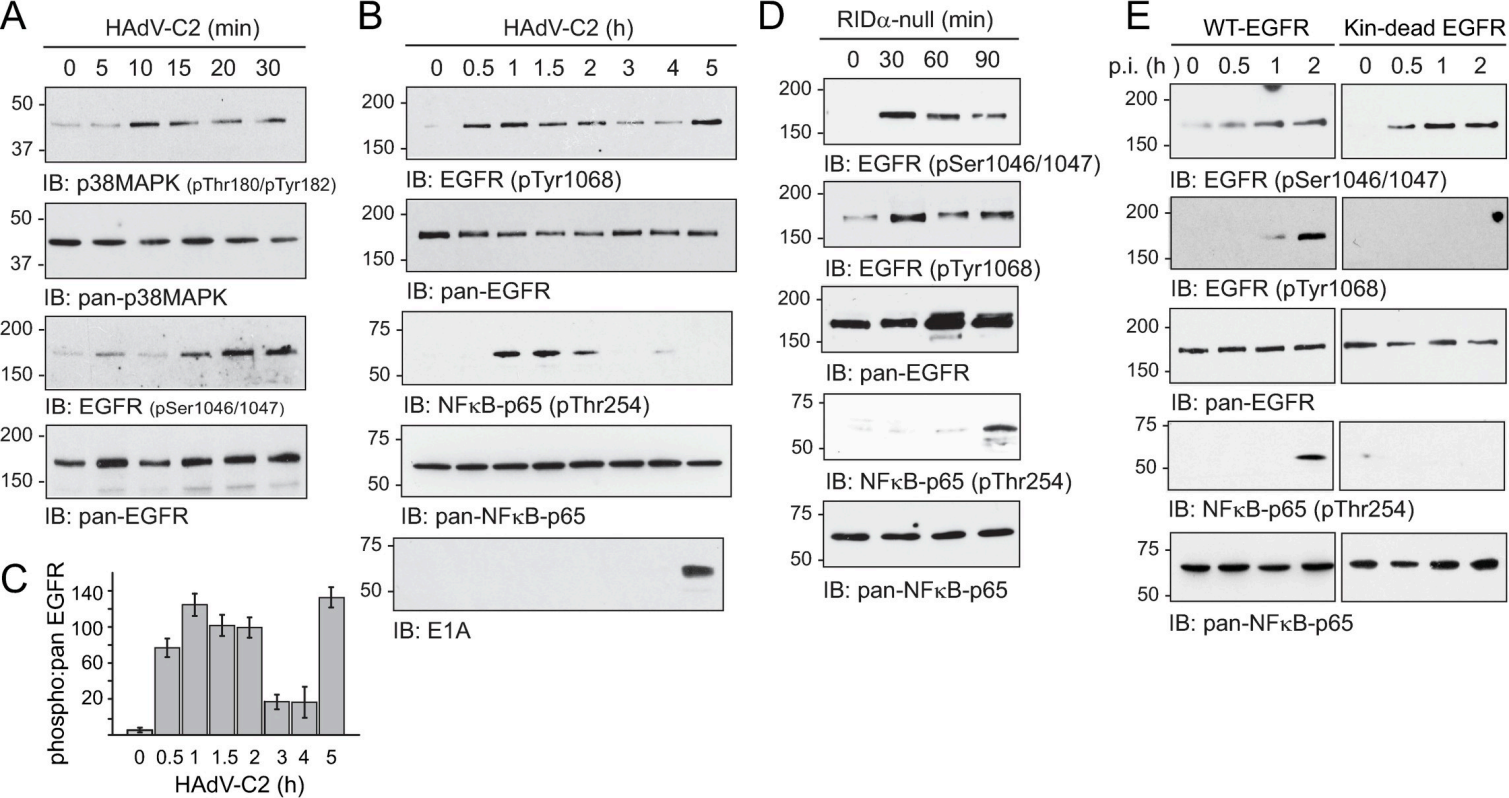
HAdV-C2 cell entry induced an EGFR/NFκB signaling axis prior to early viral protein expression. (A) A549 cells were harvested under basal conditions or every 5–10 min following an acute HAdV-C2 infection (MOI = 100). Equal protein aliquots were immunoblotted with phospho-specific and pan antibodies to p38-MAPK and EGFR. (B and C) A549 cells were harvested under basal conditions, or periodically up to 5 h following an acute HAdV-C2 infection (MOI = 100). Equal WCL aliquots were immunoblotted with phospho-specific and pan antibodies to EGFR and NFκB-p65, and to E1A (B). The amounts of the phospho-EGFR bands were quantified relative to the pan-EGFR loading control in (B) (mean ± s.e.m., n = 2) (C). (D) A549 cells were harvested under basal conditions, or periodically following an acute infection with the RIDα-null virus (MOI = 100), for immunoblot analysis with phospho-specific and pan antibodies to EGFR and NFκB-p65. (E) EGFR-null mouse fibroblasts reconstituted with WT or kinase-dead EGFR were infected with HAdV-C2 (MOI = 250), for analysis with phospho-specific and pan antibodies to EGFR and NFκB-p65. Experiments were repeated two to three times and representative results are shown.

Although NFκB-p65 was known to be activated by viral cell entry in alveolar epithelial cells, the upstream signaling mechanisms regulating this important pro-inflammatory pathway are incompletely understood [33,35,36,37]. Based on results in TNF-α-stimulated cells, we tested whether NFκB-p65 phosphorylation was regulated by EGFR signaling as a stress response to HAdV-C2 infection. Confirming reports in the literature, p38-MAPK was rapidly activated in cells infected with HAdV-C2 at a multiplicity of infection (MOI) of ~100 particles/cell (Fig 6A) [83]. HAdV-C2 infection also led to rapid EGFR phosphorylation at residues Ser1046/1047, which are substrates for the p38-MAPK effector MK2 (mitogen-activated protein kinase-activated protein kinase 2) that is known to be activated by viral cell entry (Fig 6A) [83,84,85,86]. We also found that EGFR was autophosphorylated at Tyr1068 several hours before the adenovirus E1A gene product was expressed (Fig 6B). Similar to results observed in TNF-α-stimulated cells (see Fig 5A), the EGFR activation profile was biphasic (Fig 6B and 6C). In addition, HAdV-C2 infection triggered NFκB-p65 Thr254 phosphorylation prior to E1A gene product detection by immunoblotting (Fig 6B) [54,87]. To rigorously rule out a role for the RIDα protein in these early responses, we showed that the RIDα-null mutant virus led to the same site-specific EGFR and NFκB-p65 phosphorylation events within the first 90-min of infection (Fig 6D). Since conventional tyrosine kinase inhibitors may not be effective towards transactivated EGFRs [43], we used a genetic approach to verify a role for stress-induced EGFR activity in NFκB-p65 signaling induced by adenovirus infection. These studies were carried out in EGFR-null mouse fibroblasts that were reconstituted with WT-EGFR, or a kinase-dead EGFR mutant that we had shown previously was down-regulated in adenovirus-infected cells similar to the WT receptor [19]. Although both receptor proteins were phosphorylated at Ser1046/1047 shortly after viral infection, EGFR underwent stress-induced autophosphorylation in cells expressing WT but not kinase dead-EGFR (Fig 6E). In addition, the phospho-Thr254 NFκB-p65 modification was only observed in infected cells expressing WT-EGFR, supporting the hypothesis that EGFR signaling was the upstream pathway regulating Thr254 phosphorylation (Fig 6E).

### Effect of different adenovirus serotypes

**Fig 7:**
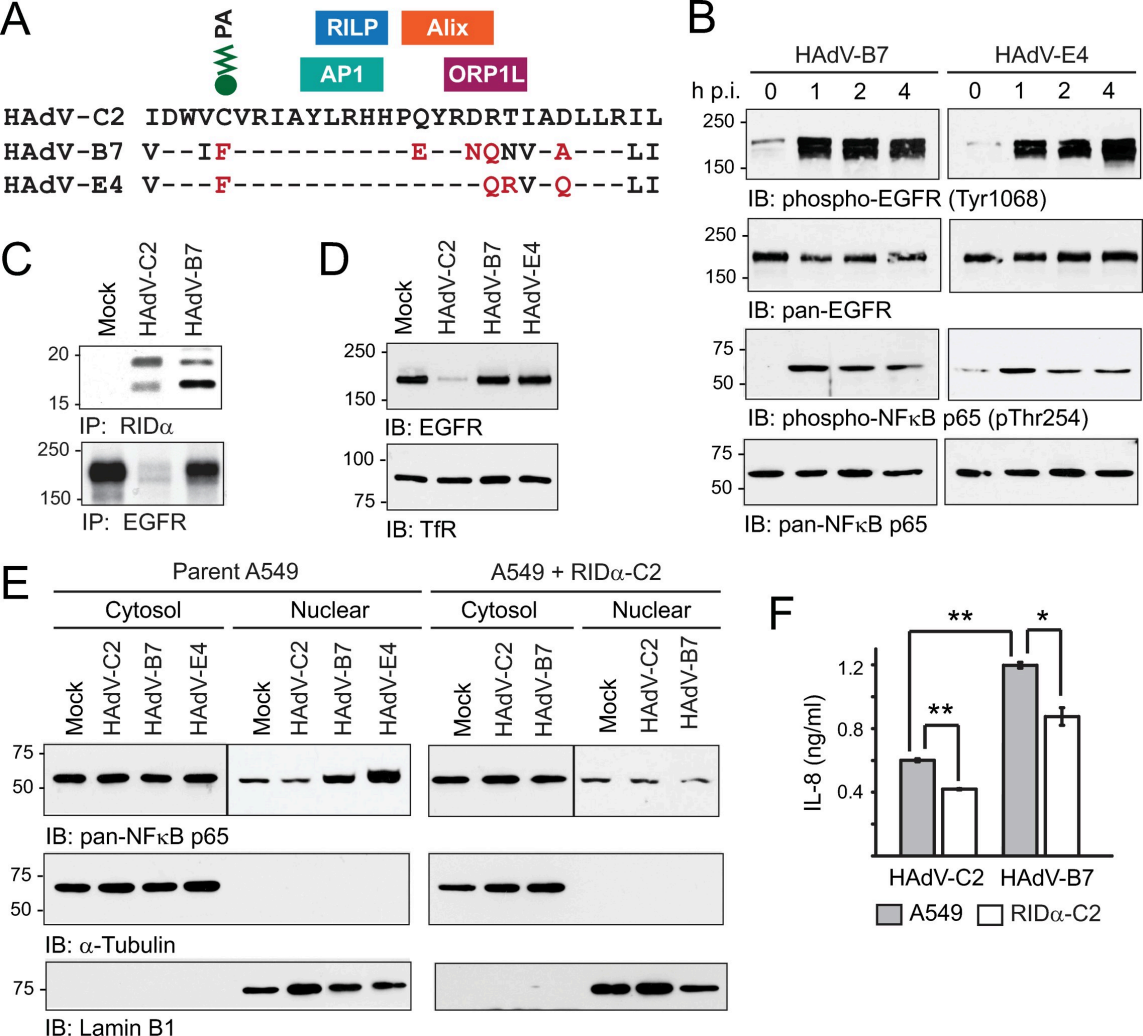
Analysis of EGFR/NFκB signaling axis after infection with different adenovirus serotypes. (A) Sequence alignment of published RID-α cytosolic tail sequences from adenovirus serotypes HAdV-C2, HAdV-B7, and HAdV-D4. HAdV-C2 RIDα is known to interact with multiple cellular proteins including Alix and RILP discussed in this study, AP1 clathrin adaptor, and the oxysterol binding protein ORP1L [52,77]. HAdV-C2 RIDα is also reversibly modified with palmitic acid (PA) at Cys67 [89]. Divergent amino acids in HAdV-B7/D4 affecting the Cys67 palmitoylation site, or introducing negative/positive to uncharged amino acid substitutions, are highlighted in red. (B) A549 cells were infected with HAdV-B7 or HAdV-D4 (MOI = 100) for immunoblot analysis of WCL with phospho-specific and pan antibodies to EGFR and NFκB-p65. (C) Mock-treated cells and cells infected with HAdV-C2 or HAdV-B7 (MOI = 100) were metabolically labeled from 1 to 2 h p.i., and RIDα and EGFR immune complexes were detected by fluorography at 12 h p.i. (D) Equal aliquots of WCL from mock-treated and cells infected for 18 h (MOI = 100) were immunoblotted with receptor-specific (EGFR and TfR) antibodies. (E) Equal aliquots of cytosol and nuclear extracts from parent A549 and A549 + RIDα-C2 cells infected with different adenovirus serotypes (MOI = 100) were immunoblotted with antibodies to NFκB-p65, and cell fractionation markers lamin B (nuclear) and GAPDH (cytosol). (F) Tissue culture supernatants were analyzed for IL-8 protein by ELISA, 24 hr after infection of parent A549 cells and A549 + RIDα-C2 cells with HAdV-C2 and HAdV-B7 (MOI = 50) (mean ± s.e.m., n = 3). All experiments were repeated at least three times and representative results are shown.

Our previous studies have shown that RIDα-C2 has several small interaction modules composed of 2–6 residues contributing to various regulatory functions located within its C-terminal region (Fig 7A). In addition to binding motifs for Alix and RILP that have already been described, RIDα-C2 interacts with the clathrin adaptor AP1 regulating its trafficking from the *trans*-Golgi network to endosomes; and the oxysterol binding protein ORP1L responsible for coupling the viral protein to homeostatic regulatory sterol pools in the ER [52,77,88]. RIDα-C2 function has also been shown to be regulated by reversible palmitoylation at Cys67 [89]. Interestingly, pathogenic adenovirus serotypes HAdV-B7 and HAdV-E4 differ in two regards: They lack the palmitoylation site, and the Alix binding site has negative/positive to uncharged amino acid substitutions (Fig 7A). The goal of these experiments was to determine whether these divergent sequences were associated with differences in the stress-activated EGFR/NFκB-p65 signaling pathway. Similar to HAdV-C2 (see Fig 6A and 6B), HAdV-B7 and HAdV-E4 both triggered EGFR autophosphorylation at Tyr1068, and NFκB-p65 phosphorylation at Thr254, within the first hour of infection (Fig 7B). We next showed that HAdV-B7 encoded an immunologically cross-reactive RIDα protein, but failed to significantly reduce EGFR metabolic stability in contrast to HAdV-C2 (Fig 7C). Similarly, HAdV-B7 and HAdV-E4 did not have a discernible effect on total EGFR protein compared to HAdV-C2, following an 18 h infection (Fig 7D). Cells infected with HAdV-B7 and HAdV-E4 also exhibited increased nuclear accumulation of NFκB-p65 protein relative to HAdV-C2-infected cells (Fig 7E). Similar to results using TNF-α as a stress stimulus, NFκB-p65 nuclear accumulation was attenuated when A549 cells with constitutive RIDα-C2 expression were infected with HAdV-B7 (Fig 7E). We also found that HAdV-B7 triggered a significant increase in IL-8 expression that was on par with other reports in the literature (~ 1.2 ng/ml 24 h post-infection) compared to HAdV-C2 (Fig 7F) [90]. Furthermore, IL-8 production was significantly reduced in cells with constitutive RIDα-C2 expression infected with either HAdV-C2 (**P < 0.0001) or HAdV-B7 (*P = 0.0206) (Fig 7F). Our results supported a hypothesis that stress-induced EGFR signaling, and its serotype-specific antagonism in HAdV-C2, are important factors in shaping the epithelial cell response to adenovirus infections.

### RIDα was a partial Rab7 mimic

**Fig 8:**
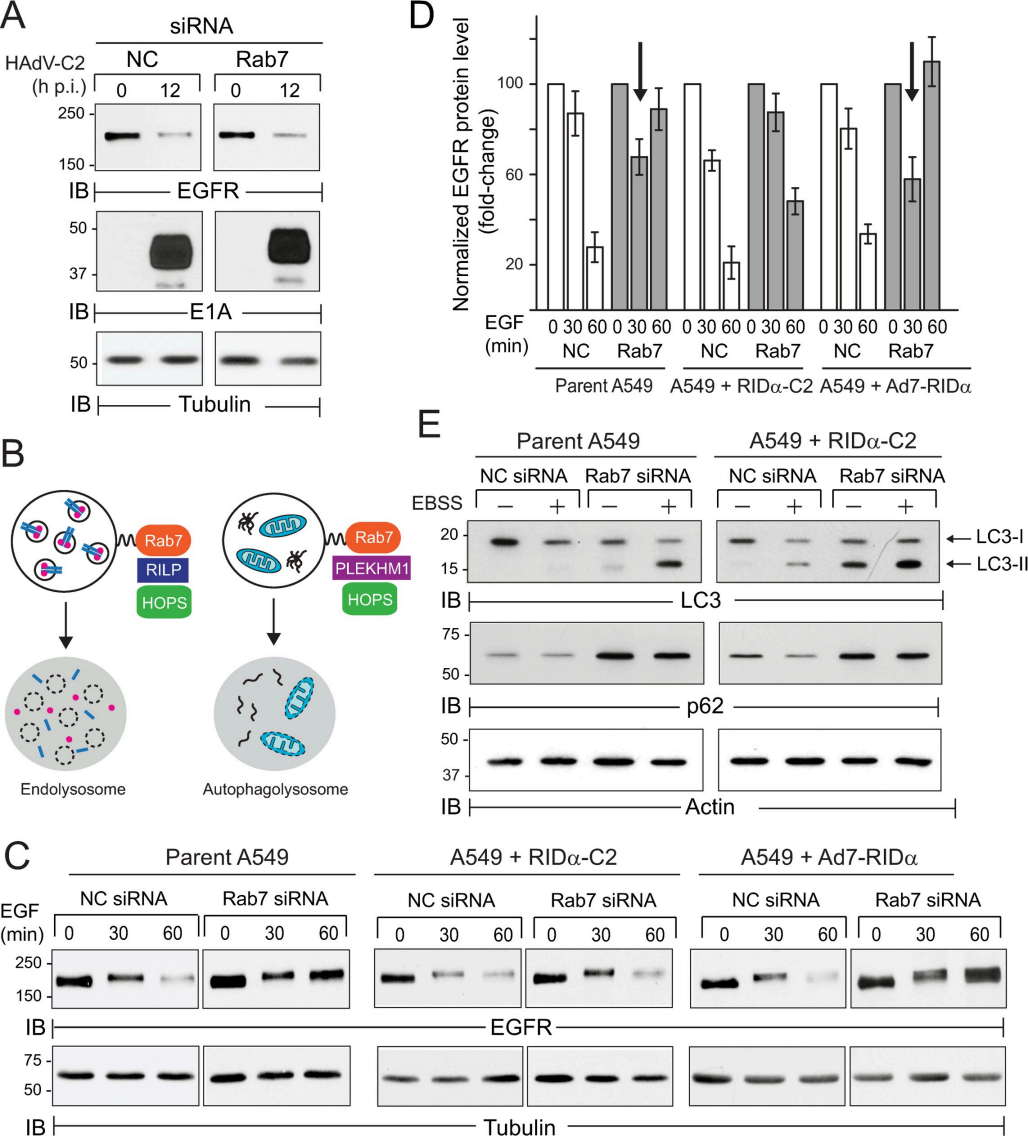
RIDα-C2 was a partial mimic for Rab7. (A) Cells treated with NC or Rab7-specific siRNAs were infected with HAdV-C2 (MOI = 100) for analysis of total EGFR and E1A protein by immunoblotting. (B) Diagram illustrating that Rab7 is linked to HOPS fusion machinery by different mechanisms in endocytic and autophagy pathways [112,129]. (C and D) Parent A549 cell and A549 cells with constitutive expression of RIDα proteins from HAdV-C2 and HAdV-B7 (RIDα-C2 and RIDα-B7 respectively) were treated with NC or Rab7-specific siRNAs for 72 h. Equal WCL aliquots collected under basal conditions, or following stimulation with EGF (50 ng/ml) for up to 60 min, were analyzed for total EGFR protein by immunoblotting (C). Quantification of normalized EGFR protein bands in (C) are presented as fold-change relative to the band at time 0 for each siRNA treatment (mean ± s.e.m., n = 3) (D). Arrows denote an apparent reduction in EGFR protein in Rab7-depleted parent and RIDα-B7-expressing A549 cells treated with EGF for 30 min discussed in the text (D). (E) siRNA-treated cells were harvested under nutrient-rich conditions, or following starvation in amino acid-deficient Earle’s basic salt solution (EBSS) for 3 h to induce autophagic flux, which was monitored by LC3 and p62 immunoblotting. (A, C, E) Equal protein loading was verified by tubulin (B and C) or actin (E) immunoblotting.

Stress-internalized EGFRs generally recycle back to the plasma membrane when the cellular stress has resolved, implying that the RIDα protein changes the balance between EGFR recycling and degradation during stress-induced receptor trafficking [42,43]. EGFR down-regulation induced by HAdV-C2 was not impaired by Rab7 gene silencing, indicating EGFRs were not diverted to the canonical Rab7 degradative pathway in infected cells (Fig 8A). This finding was consistent with our previous results showing that adenovirus-mediated EGFR trafficking was regulated by an interaction between RIDα-C2 and RILP, which couples Rab7 to the HOPS (homotypic fusion and vacuole protein sorting) complex responsible for late endosome-lysosome fusion (Fig 8B) [77]. To determine whether the viral protein induced lysosomal EGFR sorting in the absence of other viral proteins, we tested whether constitutive RIDα expression rescued ligand-induced EGFR down-regulation in Rab7-depleted cells. Rab7 gene silencing effectively blocked ligand-induced down-regulation in parent A549 cells, but not in cells with stable RIDα-C2 expression (Fig 8C and 8D). In contrast, the RIDα-B7 protein encoded by HAdV-B7 did not reconstitute ligand-induced EGFR trafficking in Rab7-depleted cells (Fig 8C and 8D). Results in A549 cells expressing different RIDα proteins therefore supported the hypothesis that serotype-specific amino acid sequences (see Fig 7A) are key determinants of functional activity regulating EGFR trafficking. We also observed a time-dependent reduction in EGFR protein that could be extracted with non-ionic detergent, following Rab7 depletion in parent A549 cells and cells reconstituted with RIDα-B7 (arrows in Fig 8D). Although the reasons for this are not clear, we speculate that internalized EGFRs may be mis-trafficked through aberrant compartments with accumulated lipid raft components, which are not fully solubilized with non-ionic detergents [91,92]. Rab7 also regulates autophagosome maturation through a different effector molecule linking it to the HOPS tethering complex (Fig 8B). We therefore determined whether RIDα reconstituted starvation-induced autophagy in Rab7-depleted cells. Autophagic flux was assessed by examining turnover of microtubule-associated protein light chain 3 (LC3), which is converted from its cytosolic form (LC3-I) to a lipid-modified species (LC3-II) and subsequently degraded during autophagic progression [93,94,95,96,97]. Rab7 depletion was associated with a build-up of LC3-II in amino acid starved cells consistent with a block in autophagosome-lysosome fusion (Fig 8E). Rab7-depleted cells also accumulated the adaptor protein p62 that acts as a cargo receptor for targeting Ub-substrates to autophagosomes [98] (Fig 8E). In contrast to ligand-induced EGFR turnover, however, heterologous RIDα expression did not reconstitute autophagic flux of LC3 or p62 in Rab7-depleted cells (Fig 8D). In fact, stable RIDα expression led to a modest reduction in autophagic flux even under nutrient-rich conditions (Fig 8E). These results suggested RIDα-C2 altered the trafficking fate of stress-internalized EGFRs by mimicking Rab7-regulated lysosome fusion with late endosomes but not autophagosomes.

## Discussion

**Fig 9:**
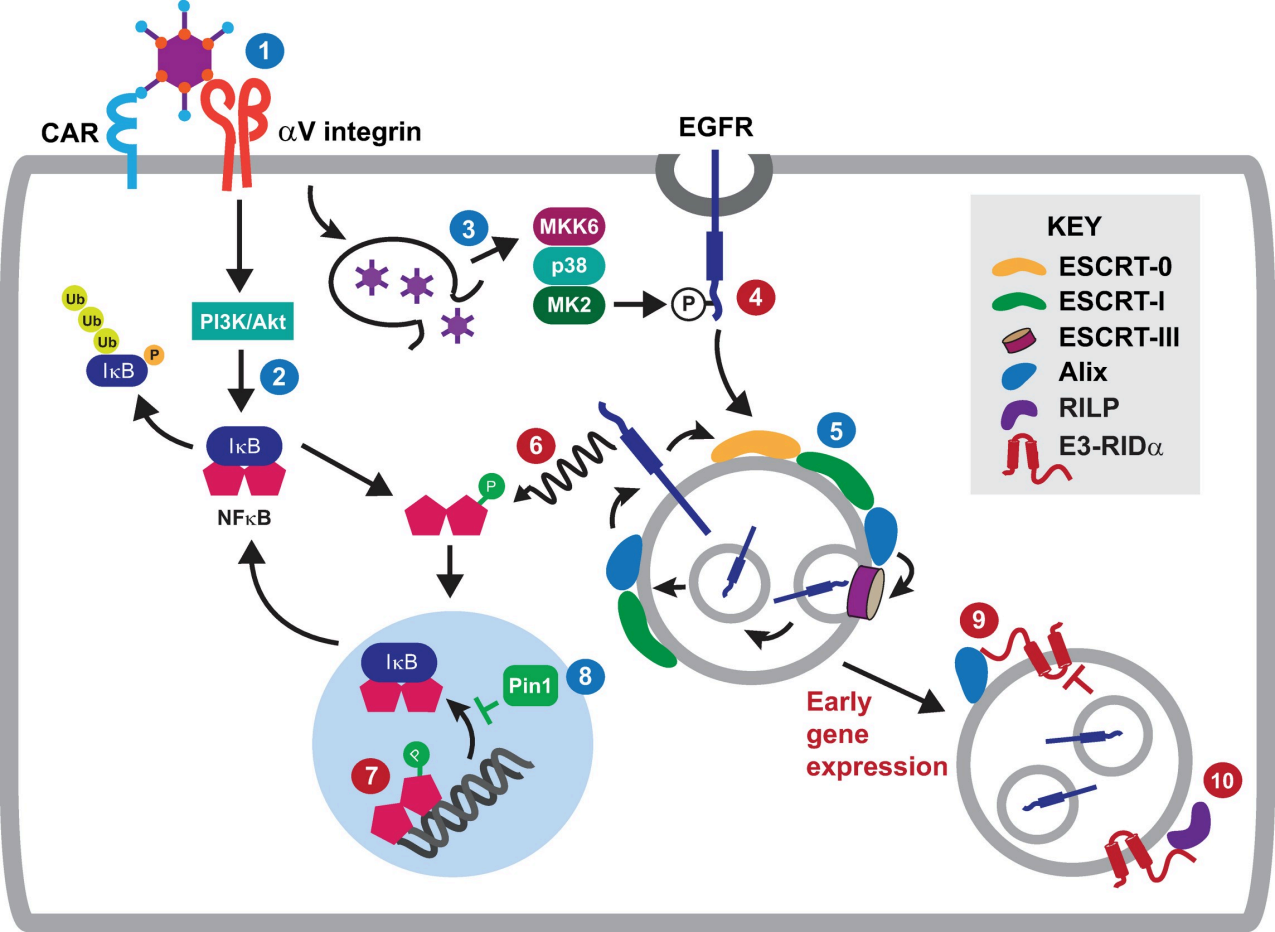
A working model for adenovirus regulation of EGFR stress responses. The following model integrates published data and results of this study (highlighted with blue and red numbers respectively). **Step 1:** HAdV-C2 internalization is mediated by sequential binding to host cell proteins CAR (coxsackie/adenovirus receptor) and αV integrins [99,101]. **Step 2:** The interaction between HAdV-C2 and αV integrins activates a PI3K/Akt signaling pathway contributing to NFκB-dependent cytokine expression through phosphorylation and consequent degradation of IκB inhibitory protein [33,104]. **Step 3:** Similar to other cell stresses, capsid escape to cytosol activates a p38-MAPK/MK2 signaling cascade [83]. **Step 4:** Adenovirus exposure induces phosphorylation of EGFR residues Ser1046/1047, which are known p38-MAPK/MK2 substrates required for stress-induced internalization from clathrin-coated pits [86]. **Step 5:** Stress-internalized EGFRs are sorted to ILVs with the capacity to back-fuse with MVB limiting membranes under regulation of Hrs, Tsg101, and Alix. **Step 6:** Adenovirus cell entry activates an EGFR signaling pathway leading to phosphorylation of the Thr254-Pro motif in NFκB-p65. **Step 7:** Stress-induced EGFR signaling is associated with accumulation of nuclear NFκB-p65 protein and enhanced NFκB gene transcription. **Step 8:** PhosphoThr254-Pro is a known substrate for Pin1, which attenuates negative feedback control of NFκB activity by preventing IκB rebinding and nuclear exit [54]. **Step 9:** RIDα-C2 attenuates stress-induced EGFR signaling from MVB limiting membranes through an interaction with Alix 3 to 4 hours post-infection. **Step 10:** The interaction between RIDα-C2 and RILP induces lysosome-mediated EGFR degradation without affecting autophagy, in the absence of functional Rab7.

Our studies have revealed three major new findings. First, incoming viral particles induced a non-canonical pathway of stress-activated EGFR trafficking and signaling prior to nuclear translocation and transcription of viral DNA in epithelial target cells. Second, stress-induced EGFR signaling was required for a site-specific phosphorylation event with a pivotal role in NFκB-p65 function. Third, the adenoviral RIDα protein attenuated the EGFR/NFκB signaling axis in the context of an acute adenovirus infection, and as an independently expressed transgene in cells stimulated with the pro-inflammatory cytokine TNF-α. The following working model interprets data presented here in the context of the current literature (see Fig 9 for a schematic diagram).

The first set of events in the working model depicts the known contribution of consecutive interactions between proteins in the icosahedral viral capsid and host cell receptors to adenovirus-directed innate immunity. The first interaction is mediated by the knob domain of fiber proteins emanating from icosahedral vertices, which bind the coxsackievirus adenovirus receptor (CAR) for all serotypes except those belonging to group B [99,100]. This is followed by a secondary interaction between a motif in the penton protein located at the base of the vertices and αV integrins [101]. It has been demonstrated that the second interaction activates a PI3K/Akt signaling pathway contributing to NFκB-dependent cytokine expression in cultured epithelial cells [33]. It is also known that membrane rupture and endosomal escape of incoming adenovirus particles activate p38-MAPK and its downstream effector MAPKAP kinase 2 (MK2), by a mechanism that is dependent on the p38-MAPK kinase MKK6, but independent of integrin-mediated cell signaling [83]. Similar to other cell stresses, our data indicated that adenovirus cell entry caused EGFR phosphorylation at known p38-MAPK/MK2 substrates (Ser1046/1047), which have previously been linked to stress-induced EGFR internalization from clathrin-coated pits [86].

The second set of events in the working model describes what is currently known about stress-induced EGFR sorting in MVBs. Previous studies have shown that stress-internalized EGFRs are sorted onto ILVs that have the capacity to back-fuse with the MVB limiting membrane, which facilitates EGFR signaling through cytosolic substrates; and eventually EGFR recycling to the cell surface upon termination of p38-MAPK signaling [44]. Stress-induced EGFR trafficking has also been shown to be regulated by a subset of ESCRT regulatory proteins Hrs, Tsg101, and Alix [44]. The requirements for Hrs and Alix were not surprising, since both of these ESCRT-associated proteins had already been linked to non-conventional endosomal sorting: Hrs through recognition of hydrophobic amino acid clusters regulating degradation of cytokine receptors [71,102]; and Alix by mediating Ub-independent ESCRT-III/MVB sorting of P2Y_1_ purinergic receptors, and through its known involvement in ILV back-fusion [71,102,103] However, a role for Tsg101 in sorting non-ubiquitinated cargo such as stress-exposed EGFR remains unclear.

The third set of events in the working model summarizes data from this study describing a novel stress-induced EGFR signaling pathway resulting in stabilization and enhanced activity of nuclear NFκB in respiratory epithelial cells. In the canonical NFκB pathway, NFκB subunits are sequestered in the cytoplasm as inactive dimers bound to inhibitory IκB proteins in resting cells [40]. Most upstream stimuli activate NFκB by inducing phosphorylation-dependent proteasomal degradation of IκB proteins [40]. Primarily regulated by inducible IKKs (inhibitor of NFκB kinases), this key step is also catalyzed by Akt, which is known to be activated by capsid engagement of αV integrins [33,104]. Liberated NFκB dimers then translocate to the nucleus, where they bind specific DNA sequences via a conserved Rel homology domain (RHD) at their N-terminus. One established mechanism for terminating NFκB responses involves newly synthesized IκB proteins induced by activated NFκB, which enter the nucleus, remove NFκB from DNA, and relocalize it to the cytosol [105]. It has also been shown that Pin1 antagonizes negative feedback control of NFκB-p65 signaling by inhibiting binding to IκBα, resulting in increased nuclear accumulation and protein stability of NFκB-p65 and enhanced NFκB activity [54,81]. Our results supported a novel hypothesis that cellular stresses, including adenovirus infection and exposure to TNF-α, contributed to sustained activation of NFκB signaling through a non-canonical EGFR pathway associated with phosphorylation of a Thr254-Pro motif in NFkB-p65, which is a known Pin1 substrate that is unmasked by IκB degradation [54]. Although the EGFR-stimulated pathway regulating proline-directed phosphorylation at Thr254-Pro motif is not currently known, our data supported a possible role for the adaptor protein Gab1. In addition to sustaining EGFR/ERK-MAPK signaling by facilitating activation of the tyrosine phosphatase Shp2, full Gab1 activity is known to require trafficking to endosomes [54,81,106,107,108]. Gab1 also links EGFRs to multiple signaling pathways including PI3K/Akt signaling cascades, suggesting stress-activated EGFRs may have additional unappreciated roles in viral replication.

The fourth set of events in the working model illustrates how RIDα-C2 attenuates stress-induced EGFR/NFκB signaling axis in the absence of other viral proteins. Our studies suggested that RIDα-C2 orchestrated a novel two step process, first by co-opting ESCRT machinery regulating stress-induced EGFR trafficking in MVBs, and then by facilitating lysosomal degradation in the absence of functional Rab7. In the first mechanism, we showed that the adenoviral RIDα protein attenuated EGFR/NFκB signaling by eliminating the stress-induced Tsg101 trafficking step in MVBs. Tsg101 reportedly controls endosome-to-cytosol release of enveloped viral RNA through an interaction with Alix, implicating a potential role in ILV back-fusion [76]. Since the viral protein formed a molecular complex with Alix, our results supported a novel hypothesis that RIDα blocked back-fusion through competitive inhibition of Tsg101/Alix binding that warrants further investigation. In contrast to the canonical Ub-dependent MVB sorting pathway where Tsg101 was required for degradative EGFR sorting, Tsg101 could have the opposite effect during stress-induced EGFR trafficking by regulating back-fusion and recycling [70]. It will therefore be of interest to determine whether pathological EGFR stress signaling could be down-regulated by manipulating the Tsg101 “steadiness” box, which controls steady-state Tsg101 protein levels under normal physiological conditions [109].

In the second mechanism, RIDα-C2 promoted EGFR degradation. Although MVB sorting silences EGFR signaling by sequestering receptors away from cytosolic effectors, lysosomal degradation appears to be the rate-limiting step in receptor inactivation [110]. Lysosome fusion is regulated in part by RILP, a downstream effector of Rab7 that recruits the HOPS membrane tethering complex to late endosomal compartments via protein-protein interactions with several HOPS subunits [111]. Our prior studies revealed that RIDα-C2 interacted with RILP, and that this interaction was required for adenovirus-induced EGFR down-regulation [77]. We have now shown that Rab7 was dispensable in the adenovirus-induced EGFR trafficking pathway, and that RIDα-C2 expression rescued ligand-induced EGFR degradation following Rab7 gene silencing. However, the RIDα protein encoded by HAdV-B7 failed to reconstitute the ligand-induced pathway, underscoring the potential importance of specific amino acids in the C-terminal tail of RIDα-C2 in regulating EGFR trafficking. Despite Rab7 also having a critical role in autophagosome maturation, however, RIDα-C2 expression did not support starvation-induced autophagy in Rab7-depleted cells [94]. This may reflect the fact that HOPS recruitment during autophagy requires distinct Rab7 effector called PLEKHM1 [112].

The finding that RIDα was a partial Rab7 mimic adds to the growing complexity of molecular mechanisms and biological functions of autophagy during adenovirus infections. On the one hand, we have shown that early adenovirus gene expression and virus production were both enhanced in airway epithelial cells with elevated autophagy, suggesting viral particles were more efficiently released from early endosomes that had fused with autophagosomes [60]. Thus adenovirus and perhaps other respiratory pathogens may co-opt autophagy, which is an important adaptive response to high oxygen pressure in airway epithelia, to help overcome epithelial barrier defenses to infection [113]. Conversely, several early adenoviral transcription units, including E1A, E1B, and E4, have been implicated in the regulation of autophagic machinery [114,115,116,117]. Adenovirus-induced autophagy is thought to be critical for recycling nutrients that support viral replication, and ultimately for promoting cell lysis necessary for efficient release of new virions [114,115,116,118]. Early adenoviral transcription units act cooperatively, either by activating (E1A and E1B) or suppressing (E4) autophagy; and cross-talk between these opposing regulatory mechanisms is thought to prevent excessive autophagy leading to premature cell death before viral replication is complete. The RIDα protein may allow infected cells to calibrate adenovirus-induced autophagic flux to different stresses in the local host microenvironment that activate EGFR signaling in endosomes. RIDα-mediated trafficking could down-tune autophagic flux during the early stages of infection, by competing for a limited cytosolic pool of Rab7 and possibly other rate-limiting shared machinery regulating lysosome fusion during endocytosis and autophagy.

In summary, our results supported a working model that RIDα-C2 restored negative feedback control to NFκB signaling, by antagonizing a stress-induced EGFR pathway associated with enhanced NFκB-p65 protein stability and NFκB activity. In addition to RIDα-C2, two other E3-encoded proteins with seemingly opposing effects on NFκB signaling have been identified: the E3-19K viral protein induced NFκB activity through ER overload [39]; and the E3 protein 14.7K inhibited NFκB transcriptional activity through an interaction with the NFκB p50 subunit that blocks DNA binding [119]. Collectively, these E3 proteins may dampen the inflammatory response to group C adenoviruses, by controlling NFκB-driven release of the neutrophil chemoattractant IL-8 from infected epithelial cells [120,121]. Alternatively, E3 proteins could fine-tune combinatorial control of NFκB signaling, which seems to be important for protecting epithelial cells from inflammation caused by innate immune cells recruited to epithelial surfaces of infected cells [32]. Our studies supported future efforts to determine whether serotype-specific differences in RIDα and other E3 proteins contribute to inflammatory disease associated with HAdV-B7, which is known to induce IL-8 protein production by a mechanism requiring virus internalization but not viral protein expression [122]. Our results also suggested RIDα-C2 could limit the innate immune response to E1A-deleted adenovirus vectors.

There is increasing evidence that many viruses exploit EGFR function to facilitate their replication and antagonize host antiviral responses [123]. Until now it was generally assumed that viruses co-opted mechanisms induced by ligand-receptor interactions. Recognition that adenovirus contributed to NFκB signaling by activating a non-canonical EGFR pathway is significant because unique host proteins regulating this pathway represent novel drug targets for therapeutic development.

## Materials and methods

### Antibodies

The RIDα antibody was commercially produced in rabbits using a peptide corresponding to the C-terminal 15 amino acids in the protein encoded by the HAdV-C2 serotype (Rockland Antibodies and Assays; Limerick, PA) [49]. See Table 1 for a comprehensive list of primary antibodies used in this study. All secondary antibodies were purchased from Jackson ImmunoResearch (West Grove, PA).

**Table 1:** Antibodies used in the study.

Target	Source	Company	Catalog #
Actin	Rabbit	Sigma (St. Louis, MO)	A2066
Alix	Rabbit	Bethyl (Montgomery, TX)	A302-938A
CD63	Mouse	Novus (Littleton, CO)	NBP2-42225
E1A	Mouse	BD Biosciences (San Jose, CA)	554155
EEA1	Rabbit	Abcam (Cambridge, MA)	ab2900
EGFR	Rabbit	CST (Beverly, MA)	4405
EGFR (pSer1046/47)	Rabbit	CST	3777
EGFR (pTyr1173)	Rabbit	CST	4407
FLAG	Mouse	CST	8146
GAPDH	Rabbit	CST	5174
GST	Rabbit	Amersham (Pittsburgh, PA)	27-4577-01
Hrs	Rabbit	Bethyl (Montgomery, TX)	A300-989A
Lamin B1	Rabbit	Abcam	ab16048
LAMP1	Mouse	DSHB*	1D4B
LC3	Mouse	Enzo (Farmington, NY)	M115-3
NFκB-p65	Rabbit	CST	8242
NFκB (pSer468)	Rabbit	CST	3039
NFκB (pThr254)	Rabbit	GenScript (Piscataway, NJ)	A00468-100
p38MAPK	Rabbit	CST	8690
p38MAPK (pThr180/Tyr182)	Rabbit	CST	4511
p62	Mouse	Abnova (Walnut, CA)	H00008878
TNFR1	Rabbit	CST	3736
TfR	Rabbit	CST	13208
Tsg101	Rabbit	Abcam	ab133586
Tubulin	Mouse	Sigma	T8203
Ubiquitin	Mouse	Babco(Richmond, CA)	MMS-258R

*Developmental Studies Hybridoma Bank (DSHB) developed under the auspices of NICHD and maintained by The University of Iowa Department of Biological Sciences (Iowa City, IA)

### Cell lines and tissue culture

Adenocarcinomic human alveolar basal epithelial A549 cells purchased from ATCC (catalog number CCL-185) were authenticated utilizing Short Tandem Repeat (STR) profiling. A549 cells with stable expression of a FLAG-tagged RIDα-C2 gene from HAdV-C2 are described in [89]. The RIDα protein from HAdV-B7 (RIDα-B7) cloned in pcDNAI/Amp was used as template for a PCR reaction using forward (5’-ATCGTAAAGATCTTGATTCCTCGAGTTCTTATATTATTG-3’) and reverse (5’-CTAAGATCTCCTTAAAGAATTCTGAGAAGATCAGCTATAGTCCTG-3’) primers to amplify the RIDα-B7 open reading frame, and incorporate flanking BglII restriction sites (underlined). PCR products were digested with BglII, and then ligated to an amino-terminal FLAG epitope in the polylinker region of the pExchange2 plasmid (Stratagene, La Jolla, CA) digested with the same restriction enzyme. GP2-293 retrovirus packaging cells were transfected with the FLAG-tagged RIDα-B7 encoding plasmid using Trans-IT 293 transfection reagent (Mirus Bio). Pantropic retrovirus was generated upon subsequent transfection of drug-selected packaging cells with pVSV-G plasmid. Retrovirus-containing media was collected 48 h later and added to A549 cells, followed by G418 selection. Stable RIDα-B7 expression was verified by immunoblotting and immunostaining with FLAG antibodies. A549 cells and their derivatives were maintained in Ham’s F12 medium supplemented with 10% fetal bovine serum and 2 mM glutamine. Mouse NIH3T3 fibroblasts (established from NIH Swiss mouse embryo) expressing WT human EGFR, or a kinase-dead EGFR construct with a K721M mutation that abrogates ATP binding (gift of Axel Ulrich; Max Planck Institute of Biochemistry), were maintained in Dulbecco’s-modified MEM medium supplemented with 10% FBS and 2 mM glutamine [19,51]. CHO (Chinese hamster ovary) cells (gift of Martin Snider, Case Western Reserve University) were grown in MEM-alpha medium supplemented with 10% FBS and 2 mM glutamine.

### Viruses, plasmids, and siRNA transfections

Human adenoviruses HAdV-C2, HAdV-B7, and HAdV-E4 were purchased from ATCC (catalog numbers VR-846, VR-7 and VR-1572 respectively). A RIDα-null HAdV-C2 mutant virus that deleted 107 base pairs in the amino terminal region of the viral protein was described in [65]. Adenoviruses were propagated, and multiplicity of infection or MOI was determined by plaque assay in A549 cells, according to standard methods [124]. It has been estimated that an MOI of at least 5 to 10 is required to ensure that 100% of cells are infected in tissue culture [125]. In preliminary studies, it was established that an MOI of 50 to 100 triggered EGFR stress responses prior to the onset of viral gene transcription, and sufficient RIDα expression to counter these responses, in human A549 cells; and that an MOI of 200 to 250 induced these responses in mouse fibroblasts. The GFP-tagged Vps22 mammalian expression plasmid was a gift from Dr. Cecilia Bucci (Università di Lecce) [126]. Gene silencing studies were carried out using ON-TARGETplus Human siRNA Smart Pools from Dharmacon (Lafayette, CO) listed in Table 2, which were introduced to A549 cells using Oligofectamine Reagent exactly as described in [60].

**Table 2:** Dharmacon ON-TARGETplus human siRNA smart pools.

Gene target	Catalog number
Alix	L-004233-00
HRS	L-016835-00
Rab7	L-040859-02
TSG101	L-003549-00
Vps22	L-004695-02
Non-coding	D-001810-10

### Metabolic labeling, immunoprecipitation, immunoblotting, and immunoprecipitation

Cells were metabolically labeled with ^35^S-Express Protein Labeling Mix (2.5 mCi/ml; PerkinElmer Life Sciences, Boston, MA) diluted in methionine and cysteine-free medium. Radiolabeled cells were rinsed 3 times with PBS supplemented with 5 mM EDTA, 5 mM EGTA, and a phosphatase inhibitor cocktail (10 mM NaF, 10 mM Na_4_P_2_O_7_, and 1 mM Na_3_VO_4_) (PBS+); and lysed with 1% NP-40 in a solution of 50 mM Tris-HCl, pH 7.5, supplemented with 150 mM NaCl, the phosphatase inhibitor cocktail, and a cocktail of protease inhibitors (100 μM phenylmethylsulfonyl fluoride, 10 μg/ml aprotinin, 10 μg/ml leupeptin, 4 μg/ml pepstatin) (IP lysis buffer). Immune complexes were recovered by incubating lysates with antibodies of interest at 4°C overnight followed by 1-h incubation with protein A conjugated to Sepharose CL-4B beads, followed by extensive washing with IP lysis buffer. Radioactive proteins were eluted and resolved by SDS-PAGE for detection by fluorography using standard techniques. For Alix co-IP studies, cells were harvested by trypsinization, washed 2 times with PBS+, and re-suspended in 10 mM HEPES, pH 7.4, supplemented with 142.5 mM KCl, 0.2% NP-40, 2 mM NaVO_4_, 20 mM NaF, 10 mM CuCl_2_, and the protease inhibitor cocktail (co-IP lysis buffer). Cells were homogenized with 10 strokes in a Dounce homogenizer, and clarified lysates were incubated with a biotin-conjugated FLAG antibody for 2 h at 4°C. Lysates were then incubated with streptavidin beads overnight at 4°C, and immune complexes were washed 3 times with co-IP lysis buffer, eluted with Laemmli buffer, and resolved by SDS-PAGE to detect protein complexes by immunoblotting. Equal protein aliquots (determined by Bradford assay) of total cell lysates, or nuclear and cytoplasmic fractions prepared using a kit from Cell Biolabs (catalog number AKR-171), were resolved by SDS-PAGE for immunoblot analysis. Nitrocellulose filters were incubated with primary antibodies and appropriate HRP-conjugated secondary antibodies diluted in blocking solution supplemented with 5% evaporated dry milk, for detection by enhanced chemiluminescence (Amersham Life Sciences). Protein bands of interest and background measurements below each protein band, which were deducted from protein band values, were quantified with the ImageJ image processing program from the National Institutes of Health. Background-subtracted EGFR protein bands were normalized to background-subtracted loading controls for each sample, and data are presented as fold-change relative to the band at time 0 for each siRNA treatment.

### GST pulldown assays

GST fusion proteins with RIDα cytoplasmic tail peptide fragments were described previously in [52]. Fusion proteins were purified from BL21 competent E. coli using the Inclusion Body Solubilization Reagent from Thermo Scientific (catalog number 78115), according to the manufacturer’s instructions. Inclusion body lysates were incubated with glutathione-Sepharose beads (Amersham-Pharmacia, catalog number 17075601) overnight at 4°C with rotation followed by three washes with a solution of 50 mM Tris (pH 7.4), 10 mM MgCl_2_, 0.15 M NaCl, and 1% Triton X-100. Beads with attached fusion proteins were incubated with whole cell lysates prepared from CHO cells using IP buffer. Beads were washed four times with IP lysis buffer, solubilized with sample buffer, resolved by SDS-PAGE, and immunoblotted with Alix antibody to detect bound proteins.

### Confocal microscopy

Cells were seeded on glass cover-slips coated with poly-L-lysine (Sigma-Aldrich, catalog number P4707) for confocal imaging. Cells were perforated with 0.5% β-escin, which is a naturally derived saponin mixture, diluted with a solution of 80 mM PIPES, pH 6.8, supplemented with 5 mM EGTA and 1 mM MgCl_2_ for 5 min and fixed with 3% paraformaldehyde–PBS for 15 min as described previously [51]. Non-specific binding was blocked with 5% normal serum from the host animal used to generate the secondary antibody (Jackson ImmunoResearch Laboratories; West Grove, PA). Cells were stained with primary or secondary antibodies overnight at 4°C or 1 h at room temperature. Antibodies were diluted in PBS supplemented with 0.5% β-escin and 3% radioimmunoassay-grade BSA. Single vertical confocal images were acquired with a Zeiss LSM 510 Meta laser scanning microscope (Carl Zeiss MicroImaging, Jenna, Germany) using diode (excitation 405 nm), Argon (excitation 488 nm), and HeNe (excitation 543 and 633 nm) lasers, 40× or 100× Plan Apo NA 1.4 objectives, and Zeiss LSM software (Carl Zeiss MicroImaging, Jenna, Germany). Confocal images were overlayed with phase contrast images to draw cell and nucleus outlines using graphics software. Quantification of co-localization was performed by measurement of Mander's coefficient in at least 10 cells per experiment using ImageJ.

### Immunoelectron microscopy

Infected cells were rinsed once with chilled serum-free Dulbecco's modified MEM, incubated with a monoclonal antibody EGFR1 directed towards an external EGFR conjugated to 10-nm colloidal gold particles (Electron Microscopy Sciences) by the tannic acid procedure for 1 h and then re-cultured at 37°C for 30 min [19,127]. Cells were fixed with a solution of 2.5% glutaraldehyde, 2% formaldehyde, and 0.1 M sodium cacodylate, pH 7.4, for 15 min on ice, rinsed with 0.1 M sodium cacodylate, pH 7.4, and post-fixed with 1% osmium tetroxide for 1 h on ice. Fixed cells were treated overnight at 4°C with 1% uranyl acetate, rinsed three times with water, dehydrated with ethanol, and embedded in polybed resin for 3 days before being baked at 60°C. Thin sections (80-nm) were mounted on Formvar nickel-coated grids, and cells were counterstained with Reynold's lead citrate and 2% uranyl acetate and then examined on a JEOL 100 CX electron microscope. A total of 50 cells were examined for distribution of gold particles located on MVB limiting membrane or on ILVs, in two independent experiments. The means of the total gold particles localized in MVB compartments were compared using a standard two-tailed Student's *t* test.

### IL-8 ELISA

Tissue culture supernatants were collected from A549 cells following various treatments, and stored at -80°C. IL-8 ELISA was performed using an anti-IL-8 neutralizing monoclonal primary antibody, biotinylated anti-IL-8 polyclonal secondary antibody and recombinant human IL-8 protein standards (Boster Biological Technology, catalog number EK0413). The plates were developed using avidin-horseradish peroxidase conjugate and TMB substrate, and absorbance read at 450 nm using a BioTek Synergy HT instrument.

### Statistical analyses and image preparation

Statistical analyses were performed using the Student’s *t* test. *P*-values < 0.05 were considered to be statistically significant. Computer-generated images were minimally processed, and all processing was applied equally to all parts of each image as well as controls, using Adobe Photoshop CS5.1 software package. Figures were prepared with Adobe Illustrator CS5.1 software package.
